# Slipping through the Cracks: Linking Low Immune Function and Intestinal Bacterial Imbalance to the Etiology of Rheumatoid Arthritis

**DOI:** 10.1155/2015/636207

**Published:** 2015-03-12

**Authors:** Kuniaki Terato, Christopher T. Do, Hiroshi Shionoya

**Affiliations:** ^1^Chondrex, Inc., 2607 151st Place NE, Redmond, WA 98052, USA; ^2^Asama Chemicals Co., Ltd., 20-3 Nihonbashi Kodenmacho, Chuo-ku, Tokyo 103, Japan

## Abstract

Autoimmune diseases (ADs) are considered to be caused by the host immune system which attacks and destroys its own tissue by mistake. A widely accepted hypothesis to explain the pathogenic mechanism of ADs is “molecular mimicry,” which states that antibodies against an infectious agent cross-react with a self-antigen sharing an identical or similar antigenic epitope. However, this hypothesis was most likely established based on misleading antibody assay data largely influenced by intense false positive reactions involved in immunoassay systems. Thus reinvestigation of this hypothesis using an appropriate blocking agent capable of eliminating all types of nonspecific reactions and proper assay design is strongly encouraged. In this review, we discuss the possibility that low immune function may be the fundamental, common defect in ADs, which increases the susceptibility to potential disease causative pathogens located in the gastrointestinal tract (GI), such as bacteria and their components or dietary components. In addition to these exogenous agents, aberrations in the host's physical condition may disrupt the host defense system, which is tightly orchestrated by “immune function,” “mucosal barrier function,” and “intestinal bacterial balance.” These disturbances may initiate a downward spiral, which can lead to chronic health problems that will evolve to an autoimmune disorder.

## 1. Introduction

The pathogenesis and disease causative antigen(s) of autoimmune diseases (ADs) such as rheumatoid arthritis (RA) remain elusive regardless of extensive studies on potential disease causative agents such as the herpes virus gp110 protein [1], heat shock proteins [2], bacterial superantigens [3], and dietary proteins [4, 5]. The search for a disease causative antigen has mainly been based on the longstanding molecular mimicry hypothesis, which states that an exogenous substance produced or possessed by an infectious agent may share sequence or structural similarities with a self-antigen [6, 7]. Based on this hypothesis, numerous studies on antibody responses to autologous components and exogenous pathogenic agents were conducted using immunoassay systems such as ELISA and RIA. Unfortunately, these assay systems were used without recognizing a number of vexing phenomena such as intense false positive reactions caused by hydrophobic binding of immunoglobulin in sample specimens to plastic surfaces as previously reported [8–10]. Thus, we propose here a systematic reinvestigation of this theory using an appropriate assay system, which utilizes a proper blocking agent capable of eliminating all types of nonspecific reactions, as well as proper experimental design that includes antigen noncoated wells to determine the remarkably high nonspecific background noise reactions unique to individual samples [8, 10].

One candidate group of disease causative pathogens is pathogenic intestinal bacteria, which carry pathogen associated molecular patterns (PAMPs) on their surfaces, which are recognized by pattern recognition receptors (PRRs) such as Toll-like receptors (TLRs), and may activate host innate and adaptive immune responses, thus potentially triggering an uncontrollable inflammatory reaction leading to the development of ADs [11–14].

In addition to pathogenic intestinal bacteria, nonpathogenic commensal bacteria and their cellular components may also be involved in the pathogenesis of ADs. Normally, these intestinal bacteria do not affect the host's health but may overcome the host's defenses and exert pathogenic effects under conditions such as immunosenescence, GI disorders, or other events such as physical and psychological stress [15–22] as we will discuss later. Although none of these hypotheses have been confirmed, advanced technologies such as genomic analysis of bacteria [23, 24] and gnotobiological methods [25] are facilitating studies on the potential linkage or association of intestinal bacteria with ADs [13, 14, 26, 27].

Based on our previous studies [5, 10, 28–32] and others [33, 34], we propose here that the imbalance of intestinal bacteria and a consequent increase in toxin levels in the GI tract may directly contribute to the development of ADs in conjunction with low immune function at the gut associated lymphoid tissue (GALT) and an increased mucosal permeability. As a consequence, chronic translocation of excess amounts of disease causative pathogen(s), especially bacterial toxins, into the body through the mucosal barrier system might contribute to the development of inflammatory diseases, which are characterized as progressive and persistent.

### 1.1. Core Contributing Factors to Autoimmune Diseases

To understand the pathogenesis of ADs, it is important to consider that there is a gender bias in RA and type I diabetics. In fact, recent studies in mouse models suggest that higher susceptibility to arthritis [35] and type I diabetes [36] in female mice could be directly attributed to commensal bacteria. Importantly, intestinal bacteria are influenced by MHC haplotypes, and the guts of arthritis susceptible HLA-DRB1^*^0401 transgenic mice are dominated by a* Clostridium*-like bacterium, whereas the guts of arthritis resistant DRB1^*^0402 mice are enriched for members of the Porphyromonadaceae family and* Bifidobacterium* [35]. Moreover, older female ^*^0401 mice exhibit increased intestinal mucosal permeability compared to young ^*^0401 females or old ^*^0402 females. Accordingly, cytokine transcripts in jejuna showed differential TH17 regulatory network gene transcripts in ^*^0401 and ^*^0402 mice. Similarly, Markle et al. [36] show that the commensal microbiota could reinforce the gender bias in the sensitivity of female mice versus resistance of male mice to type 1 diabetes mellitus and alter serum testosterone levels. These observations indicate that intestinal flora linked to gender, age, and genetic background may modulate the gut immune system and enhance proinflammatory conditions in susceptible individuals.

Indeed, RA mainly develops in women older than 40 and men older than 60 years old as reviewed elsewhere [37] and develops long after the emergence of preclinical symptoms such as fatigue, malaise, and diffuse musculoskeletal pain [38]. In fact, we have observed that the majority of RA patients (14/18: 78%) suffer from GI disorders such as constipation (66%) or diarrhea (11%) [32]. These observations indicate that physical and psychological health problems are present in these patients before the onset of clinical RA and highlight the importance of healthy gut function, more specifically the GI immune system.

**Figure 1:**
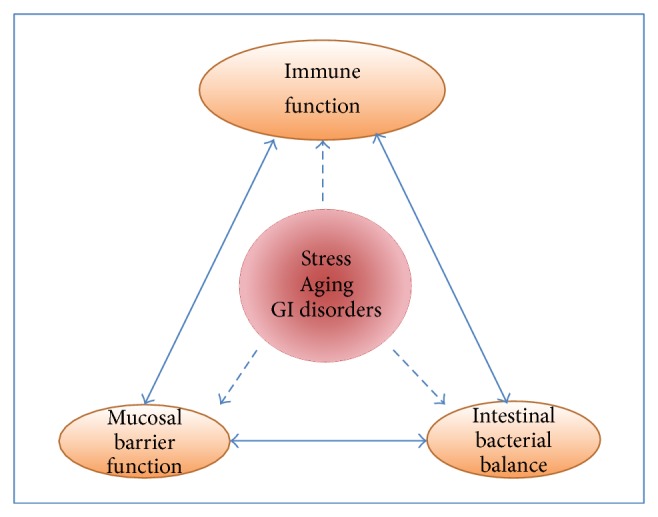
Triangle of three key elements closely linked to maintain host health: immune function is critical to maintaining the balance of these three elements. Once the host's immune function is deteriorated by stress, aging, and other events such as GI disorders, the mucosal barrier system will be deteriorated along with an imbalance of intestinal bacteria, which eventually overcome the GI immune system. As a consequence, hosts with low immune function become more susceptible to these intestinal pathogens and fall into a downward spiral of chronic health problems.

The GI immune system is exposed to large amounts of countless pathogenic and nonpathogenic foreign substances including dietary proteins and intestinal bacteria. Therefore, in the GI tract, three independent elements, “immune function,” “mucosal barrier function,” and “intestinal bacterial balance,” are tightly orchestrated to protect the host from hazardous pathogenic substances [14, 34, 39] as illustrated in Figure 1. This tight regulation is exemplified by the fact that the GI mucosa is consistently interacting with intestinal bacteria and their components, which stimulate the immune system and induce a state of “controlled physiological inflammation” [40] under healthy conditions. However, it is likely that mucosal barrier function is susceptible to a variety of events such as stress [41–43], GI disorders [44], and immunosenescence associated with aging [45–47], which deteriorate the host immune function and disrupt intestinal bacterial balance. As a consequence, excess amounts of exogenous substances, which may overwhelm the mucosal barrier, penetrate into the mucosa and cause a shift from a controlled inflammation to a pathological inflammation. This phenomenon is similar to the age-related proinflammatory state as characterized by higher levels of interleukin- (IL-) 6, IL-1 receptor antagonist, IL-18, and C-reactive proteins in the elderly with low immune function due to the immunosenescence [48].

In this review, we discuss a possible etiology of RA based on the hypothesis that high intestinal mucosal permeability and imbalance of intestinal bacteria associated with low immune function [10, 49] may be critical disorders in ADs such as RA. We also comment on the legitimacy of molecular mimicry as a mechanism for autoimmunity.

### 1.2. Molecular Mimicry in the Search for a Disease Causative Antigen

Rheumatic fever is considered as a sequela of group A* Streptococcus* infection, as antibodies against this bacterium are believed to cross-react to self-components such as cardiac myosin, resulting in heart valve deterioration [50]. This concept of cross-reactivity between self- and non-self-components became the basis for the hypothesis known as molecular mimicry or the cross-reactivity hypothesis as reviewed elsewhere [6, 50] and has been expanded to explain the pathogenesis of other diseases, such as RA and ankylosing spondylitis (AS). For example, the antibody epitope on the human HLA DR molecule (EQRRAA) is shared with the bacterium* Proteus mirabilis* in RA [51–54], whereas the antibody epitope on the human HLA-B27 molecule (QTDRED or DRDE) is shared with* Klebsiella*,* Shigella*,* Yersinia*, and* Salmonella* in AS [55–58]. Accordingly, numerous studies were conducted based on this hypothesis and concluded that antibodies against a variety of potential pathogenic bacteria, such as* E. coli*,* Klebsiella pneumoniae*,* Proteus mirabilis*,* Serratia marcescens* [7, 17, 59, 60], LPS [61, 62], and* P. gingivalis* [63], were higher in ADs groups than in normal controls. For example, Tani et al. [64] reported that Japanese patients with RA have high IgG antibody titers against* Proteus mirabilis* and* Klebsiella pneumonia*, while Japanese patients with AS have high IgA antibody titers against* Klebsiella pneumonia* but not* Proteus mirabilis*, indicating that a particular strain of bacteria may be involved in different diseases. Although this proposal is very attractive for examining the possible involvement of a variety of bacteria in ADs, a reinvestigation using more appropriate assay protocols and buffer systems with careful consideration on the significant differences in the interactions between individual subclasses and isotypes of serum immunoglobulins from patients with different diseases and individual strains of bacteria is needed, as we briefly discuss below.

These kinds of data and consequently molecular mimicry as a proposed mechanism for ADs have persisted for two reasons. The first is the lack of recognition of intense false positive reactions caused by the sample itself in immunoassays, which are higher in autoimmune patients than in normal controls. In fact, this was clearly addressed by de Vries et al. [9], who ran ELISAs using two additional control wells: blank wells coated only with buffer and wells coated with an irrelevant antigen, in addition to wells coated with a synthetic HLA-B27 peptide to assay serum antibodies in patients with AS and Reiter's syndrome. Indeed, almost identical OD values were observed in all three wells due to the hydrophobic binding of immunoglobulin in sample sera to plastic surfaces of the ELISA plates. Secondly, it is not fully appreciated that human sera, regardless of normal or diseased, contain high levels of IgG and IgA antibodies against virtually all environmental agents, such as dietary proteins, bacteria, and their cellular components, all of which humans are continuously exposed to during their lifetime [10]. Most importantly, it is virtually impossible to differentiate disease groups from healthy controls by the immunoassay systems used in the past and present. This is especially true for low serum dilutions (1 : 20–1 : 200), due to the intense false reactions involved in current immunoassay systems. Further discussion on eliminating false positive reactions in immunoassay systems is beyond the scope of this review but has recently been examined [10].

Regardless of these points of criticism, molecular mimicry persists as the main hypothesis for rationalizing the possible association of pathogenic microbes with the development of ADs [7]. Therefore, we believe that it is our obligation to raise questions about the accuracy and reliability of immunoassay systems such as RIA, ELISA, immunoblotting, and immunostaining and ask whether antibodies against these pathogens really cross-react to autologous components.

Nonetheless, the high cross-reactivity of anti-foreign substance antibodies with autologous components should not be disregarded. One example is the strong cross-reactivity of antibodies against heterologous type II collagen, such as chick and bovine, with autologous mouse type II collagen in mice [65, 66] and human type II collagen in RA [5, 29], which share high amino acid sequence homology of more than 85% [67]. Therefore, individual species of type II collagen share multiple common epitopes, while only one epitope is shared by bacteria and the HLA molecule as mentioned above. In fact, antibodies against dietary chick and bovine type II collagen that are cross-reactive with autologous human type II collagen likely play a pathological role in patients with RA [31]. These observations were confirmed by feeding chick type II collagen in an “oral collagen-induced arthritis model” [30]. These data suggest that more detailed studies are required to search for possible pathogenic mimic antigens, which elicit antibodies that are cross-reactive with autologous components by employing an appropriate assay system.

### 1.3. Low Immune Function in Patients with Autoimmune Diseases

**Figure 2:**
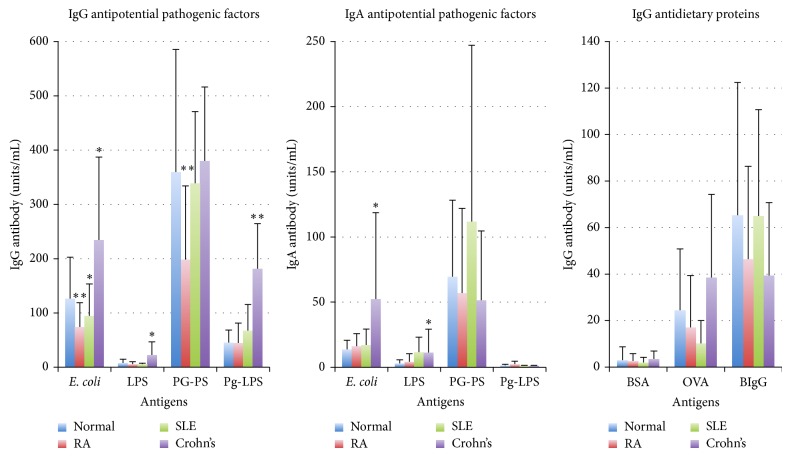
Comparison of IgG and IgA antibody responses to potential pathogenic and nonpathogenic environmental agents between normal controls and patients with RA, SLE, and Crohn's disease.

The immune system in patients with ADs was considered to be upregulated or overstimulated by autologous components or disease causative pathogens based on antibody assay data influenced by nonspecific reactions as described above. Therefore, the question of whether the host immune system is stimulated by these pathogens or otherwise lowered by the host's conditions such as immunosenescence was addressed by assaying the antibody responses to environmental agents in patients with ADs using ELISA employing a newly developed blocking agent (ChonBlock). In this study, we found that IgG antibody responses to potential pathogenic agents, such as* Escherichia coli* (*E. coli*)*, E. coli-*lipopolysaccharide (LPS), and peptidoglycan polysaccharide (PG-PS) from group A* Streptococcus pyogenes*, which is arthritogenic in experimental animals [68–70], as well as nonpathogenic dietary proteins, were significantly lower or tended to be lower in patients with RA and SLE compared to normal controls (Figure 2). On the other hand, IgA antibody responses tended to be higher, and as a consequence, the IgA/IgG antibody ratios were significantly higher in these patients. In contrast, patients with Crohn's disease displayed higher IgG antibody responses to all the antigens tested including* Porphyromonas gingivalis* (*P. gingivalis*) LPS (Pg-LPS), indicating that their immune systems are excessively stimulated by these antigens, which have penetrated into the body due to the leakage of the mucosal barrier. These observations indicate that the pathogenic mechanisms and pathogens involved in RA and Crohn's disease are apparently different as discussed later in Section 1.4.

### 1.4. Imbalance of Intestinal Bacteria Associated with Low Immune Function

**Figure 3:**
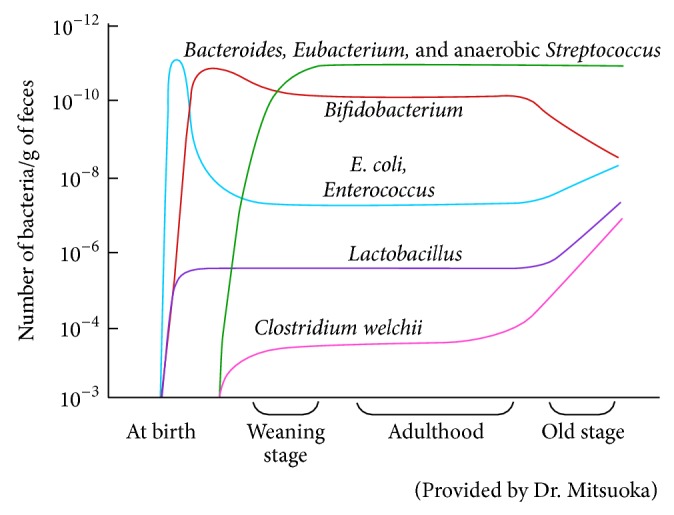
Age associated changes of intestinal flora composition.

Intestinal bacterial balance is maintained under the influence of host immune function [71, 72] from childhood through adulthood but starts to change at around 50–60 years of age [73, 74] as illustrated in Figure 3. For example, the relative population of beneficial bacteria such as* Bifidobacterium* declines gradually with aging, whereas the relative population of hazardous bacteria such as* Clostridium perfringens* (*C. perfringens*) and* E. coli* increases. This shift may be attributed to immunosenescence and may increase disease risk in the elderly [45, 73]. Similarly, the imbalance of intestinal bacteria may be due to low immune function associated with stress [41–43] and GI disorders [44]. Consequently, increases in the bacterial toxin levels in the GI tract may not only trigger and exacerbate inflammatory reactions, but also further deteriorate the host's immune system.

In fact, much evidence suggests that changes in the intestinal flora may be a possible etiopathogenic or aggravating factor in RA, and even commensal bacteria may be capable of inducing ADs. Indeed, it was reported that a vegetarian diet modulated the intestinal flora, which was associated with clinical improvement in patients with RA [16, 18, 75]. Furthermore, it was reported that certain strains of bacteria such as* Bifidobacterium*,* Bacteroides-Porphyromonas-Prevotella* group,* Bacteroides fragilis* (*B. fragilis*) subgroup, and* Eubacterium rectale-C. coccoides* group were significantly less numerous in early RA than in controls [21], indicating that these strains may be important in maintaining a barrier for the intestinal wall.

Moreover, the imbalance of intestinal bacteria in inflammatory bowel disease (IBD) has been extensively studied in humans possessing a genetic predisposition for IBD [25, 76]. Indeed, recent phylogenetic comparisons of intestinal bacteria in IBD and non-IBD controls indicated a compositional shift in the bacteria of IBD patients. More specifically, a depletion of commensals such as* Lachnospiraceae* and* Bacteroides* and an enrichment of* Proteobacteria *[77] were observed, whereas other studies indicated a high prevalence of* Bacteroides* and* Prevotella *spp. in ulcerative colitis, a type of IBD [78]. These data demonstrate the importance of intestinal bacterial composition in IBD.

Although compositional changes in intestinal bacteria are observed in patients with RA and IBD, the pathogenic mechanisms involved in RA and IBD apparently differ, as significant differences in antibody responses to environmental pathogens were observed as described in Section 1.3. The differences between RA and IBD may be explained by possible defects in innate immune function, mucosal barrier function, or T-lymphocyte regulatory function in IBD [79]. To distinguish the different types of ADs, detailed studies on possible defects in the host defense system, as well as mucosal barrier function, are required.

### 1.5. Pathogenic Role of Intestinal Bacteria in Autoimmunity

The possible involvement of pathogenic bacteria in ADs has been a prevalent focal point linking bacteria to the etiology of ADs [13, 14]. However, it has been suggested that even nonpathogenic commensal bacteria may be involved in the pathogenesis of ADs. Indeed, it is highly likely that unregulated growth of nonpathogenic commensal bacteria triggers overactivation of innate and adaptive immune responses without precise control by the host immune system [15, 17–22, 25].

For example, the glucose 6-phosphate isomerase (GPI) specific T-cell transgenic K/BxN mouse develops attenuated arthritis in germ-free conditions but develops more severe arthritis when inoculated with commensal bacteria [80]. Moreover, human B-27 transgenic rats [81] and mice [82], developed for studying the contribution of genetic background to developing ankylosing spondylitis (AS), do not develop inflammatory intestinal or peripheral joint disease in germ-free conditions. However, B-27 transgenic rats developed diarrhea and inflammatory changes in the colon after being transferred to a conventional room for 23 days. Similarly, B-27 transgenic mice showed a high incidence of paralysis and mortality following infection with* Yersinia enterocolitica*. Furthermore, the prevalence of the naturally occurring joint disease, ankylosing entheropathy, is higher in conventionally housed mice than their specific pathogen-free (SPF) counterparts [83]. This increased susceptibility to pathogenic and nonpathogenic bacteria in germ-free or SPF housed mice is attributed to an immature immune system. Thus, inoculated intestinal bacteria are capable of invading into the intestinal mucosa and can induce inflammatory reactions at the GI mucosa, as well as other tissues. These observations indicate the importance of the mucosal immune system, which is exposed to a large number of intestinal bacteria and other exogenous substances.

### 1.6. Mucosal Permeability Associated with Low Immune Function

In addition to immune function and intestinal bacterial balance, the third element that is critical to maintaining host health is the mucosal barrier function. Importantly, the mucosal barrier is not completely impermeable, as undigested dietary proteins are absorbed even in healthy normal adults [84]. Moreover, the GI mucosa is susceptible to psychological [41–43] and physical stress [85], nonsteroidal anti-inflammatory drugs [86], and GI disorders [44]. All of these events may increase the mucosal permeability to large molecules such as undigested proteins, bacteria, and bacterial cell components including toxins such as LPS. Indeed, rats under stress exhibit increased translocation of horseradish peroxidase (HRP) and* E. coli* by 4 and 30 times compared to control rats [42]. Similarly, viable gut commensal bacteria were detected in the lymph nodes, spleen, liver, and kidneys of immunodeficient mice [87, 88], suggesting the importance of immune protection against the translocation of macromolecules at the GI mucosa. Finally, degradation products of bacterial cell walls and nucleic acids have been found in joints of RA patients [19], indicating high permeability of the intestinal mucosa in these patients. In fact, some of these bacterial cell components such as a streptococcal proteoglycan polysaccharide (PG-PS) are known to be arthritogenic and capable of inducing arthritis in rodents [68–70], suggesting that bacteria and their components may play a pathological role in humans.

Furthermore, high mucosal permeability along with significant changes in the colonic flora is associated with constipation. Although constipation is not considered as a serious GI disorder, it is still considered as a risk factor for increased susceptibility to pathogenic bacteria and immune disorders [44]. In these patients, (1) serum ovalbumin (OVA) levels are 28 times, (2) anti-*E. coli* antibody levels are 9 times, (3) spontaneous lymphocyte proliferation is 1.5–2.0 times, and (4) serum IgG levels are 1.3 times higher than normal controls, whereas T-cell responses to PHA, a T-cell mitogen, are less than 1/2 of normal controls. These data indicate that increased mucosal permeability, which may be associated with low immune function, is a critical disorder linked to chronic health problems.

### 1.7. Pathological Roles of Bacterial Toxins

LPS is a major component of the outer membrane of gram-negative bacteria representing one of the conserved microbial structures. Interestingly, LPS serves unique functions for the host; one is to stimulate and mature the host immune system, while the other is to trigger and enhance inflammatory reactions. For example, the pathological role of LPS has been demonstrated, as elevated serum LPS levels were observed in patients with type I diabetes [89] and chronic fatigue syndrome [61] and even in healthy men [90, 91]. Moreover, intestinal absorption of LPS is significantly increased by fats such as triolein (olive oil) [92]. In fact, high-fat content meals induce low-grade endotoxemia in an obesity and insulin resistant mouse [93].

**Figure 4:**
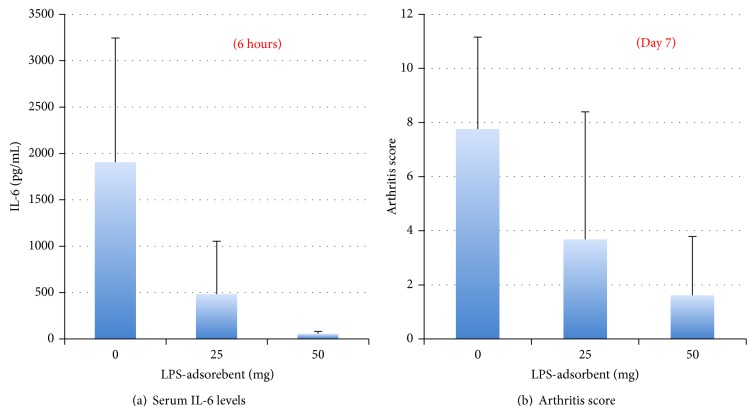
Synergistic effect of bacterial toxins and autoantibodies to type II collagen in induction of arthritis in mice. Oral administration of LPS in mice receiving a subarthritogenic dose of monoclonal antibody cocktail displayed an immediate increase in serum IL-6 levels and induced arthritis within 24–48 hours, reaching a maximum score on days 7-8. Co-oral administration of LPS-adsorbent prevents the increase of serum IL-6 and development of arthritis in a dose-dependent manner.* Note*. Serum IL-6 level was increased only in old mice more than 8 months but was not observed in young mice.

Most importantly, LPS absorbed from the intestine is capable of triggering inflammatory reactions. For example, oral administration of LPS (1 mg) into DBA/1 mice older than 1 year old, which previously received a subarthritogenic dose (1 mg) of monoclonal antibody cocktail [66], increased the serum IL-6 levels (Figure 4(a)) and induced severe arthritis (Figure 4(b)), whereas oral administration of an LPS-adsorbent prevented the increase in serum IL-6 and development of arthritis in a dose-dependent manner. Importantly, young DBA/1 mice (8–14 weeks old) did not respond to oral LPS administration, as serum IL-6 levels were not raised in these animals, indicating that aged mice are more susceptible to bacterial toxins present in the GI tract. This observation supports a possible link between immunosenescence and an age-related proinflammatory state [48], as well as the increased incidence of RA in the elderly [37].

Furthermore, oral coadministration of a low dose of LPS (10 *μ*g/mouse/day) with chick type II collagen (CII) in DBA/1 mice enhanced not only antibody production, but also CII-specific T-cell responses [30]. Moreover, mice receiving a long-term oral administration of LPS alone developed non-antibody-mediated arthritis, which was characterized by significant deformity of digit and loss of nail, indicating that LPS alone is capable of inducing chronic arthritis without autoantibodies to type II collagen. This evidence supports the observation that chronic stimulation of B cells by mitogenic LPS increased IgG secreting splenocytes, hypergammaglobulinemia, and antibody production, as well as accelerating the development of SLE in MRI/n, BXSB, and NZW female mice [101].

In addition, the proinflammatory effects of LPS and other bacterial toxins have been extensively studied in various types of animal models such as collagen-induced arthritis (CIA) [102], collagen antibody-induced arthritis (CAIA) [28], experimental autoimmune encephalomyelitis (EAE) [97], SLE-nephritis [101, 103], autoimmune thyroiditis [104], autoimmune hemolytic anemia [105, 106], and obesity [93]. Importantly, bacterial toxins, regardless of their B- or T-cell specificities, are not only lethally toxic, but capable of triggering and exacerbating inflammatory reactions. For example, T-cell mitogens, SEB [99, 107], and MAM [98] also trigger and exacerbate severe inflammatory arthritis in CIA and CAIA models, as well as LPS, a B-cell mitogen.

However, bacterial toxins not only are stimulatory, but may even diminish the host immune system [108, 109]. Indeed, T-cell responses to plant mitogens such as PHA, pokeweed, and concanavalin A (ConA) were diminished in patients with RA and constipation [110–112]. These data indicate that B and T cells polyclonally activated by mitogenic bacterial toxins absorbed from the GI tract are no longer responsive to pathogens. Taken together, chronic disturbances of the immune system by intestinal bacterial toxins appear to be an important etiology linked to low immune function and contribute to the development of ADs such as RA.

### 1.8. New Approach for Modulating Intestinal Flora and Disease Activities

Low immune function, compromised mucosal barrier integrity and intestinal bacterial balance may be critical factors in the etiology of RA and other ADs. Thus, one therapeutic approach is to restore the intestinal bacterial balance using antibiotics; however, this approach is not effective for the treatment of infectious diarrhea [95]. On the other hand,* C. difficile*-associated diarrhea, an emerging complication associated with the use of systemic antimicrobials, has been successfully treated using donor stool to normalize the microbiota [113]. Alternatively, passive antibody therapy using bovine colostrum obtained from cows immunized with specific pathogens was used to treat diarrhea in patients infected with immunodeficiency virus [114, 115] and other infectious agents such as oral candidiasis,* Helicobacter pylori*, rotavirus,* Campylobacter jejuni*, and* C. difficile* [116–118]. To confirm the beneficial effects of milk antibodies, Iwatsuki et al. [119] recently analyzed intestinal bacterial flora in elderly volunteers treated with oral administration of a natural milk antibody product, which contains active antibodies against a wide range of pathogenic bacteria and their toxins [120]. After 8 weeks of treatment, the population of* E. coli*,* C. difficile*, and* C. perfringens* (formerly known as* C. welchii*) decreased significantly, whereas* Lactobacilli*,* Bacteroides *spp.,* Prevotella *spp.,and* B. fragilis* populations increased.

This approach was further applied for the treatment of patients with RA, whose disease activity was uncontrolled by authentic medications [32], resulting in significant reduction of arthritis symptoms and improvement of intestinal disorders in 50% of 18 patients, whereas none of the 20 untreated patients showed improvement in either arthritis or GI symptoms, such as constipation and diarrhea. To confirm the efficacy of this milk antibody product, a multicentered double blind clinical study is currently being conducted. Although not yet mainstream, these alternative medical approaches for the treatment of infectious diseases are gaining traction, as more complications associated with antibiotic resistant bacteria are emerging. Moreover, we are optimistic about these approaches for the successful treatment of ADs.

## 2. Discussion

ADs such as RA may begin with marked systemic symptoms, including fatigue, fever, and weight loss, and slowly progress to classic symptoms such as joint pain and swelling. This progression suggests that a long-term exposure to disease causative pathogens might be the fundamental etiology of RA. In this aspect, we believe that various types of exogenous and internal factors listed in Table 1 are involved in developing health problems, which evolve to progressive, chronic diseases. With regard to mimic antigens, antibodies to heterologous type II collagen apparently contribute to the pathogenesis in the majority of patients with RA and relapsing polychondritis (RP) [5], whereas patients with HLA-DR haplotypes of 0401/0405, 0404, and 0408 are unlikely mediated by autoantibodies [31], but instead mediated by environmental agent(s) such as bacteria and their components, which are chronically absorbed from the GI tract over long periods.

Based on these observations, it is reasonable to consider that host factors such as immunosenescence, stress, and GI disorders, which lower the host's immune function, may play an initial role in disrupting the triangle relationship between the three key elements: “immune function,” “mucosal barrier function,” and “intestinal bacterial balance.” Accordingly, low immune function, which lowers the mucosal defense function in the GI tract, may be the primary and common defects that increase the mucosal permeability and trigger an imbalance in intestinal bacteria, which might increase the toxin levels in the GI tract.

Under these circumstances, nonpathogenic and pathogenic commensal bacteria and their toxins such as LPS may equally contribute to the pathogenesis of ADs. Currently, advanced technologies are available to analyze the complex intestinal bacterial world, and it seems that the time is ripe to study the contribution of intestinal bacteria and their cellular components in order to reveal the etiologies of RA and other chronic diseases.

## 3. Conclusion

**Figure 5:**
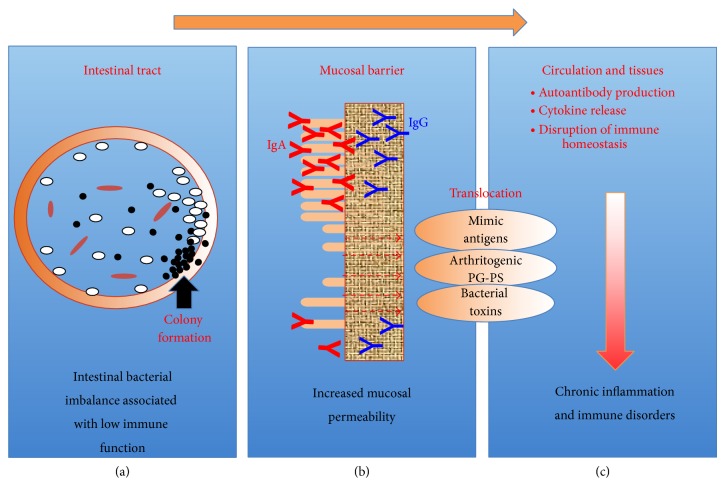
Possible contribution of pathogenic and nonpathogenic environmental factors to autoimmune diseases: low immune function may lead to intestinal bacterial imbalance by failing to prevent the colony formation of pathogenic bacteria (a). In addition decreases in the mucosal barrier function result in increased intestinal mucosal permeability (b). As a result, excess amounts of bacterial components and their toxins and even large molecular, undigested dietary proteins, which share similar amino acid sequences with autologous components such as collagen, cross the mucosal barrier into circulation (c). Some of these exogenous substances can disturb immune homeostasis and result in the development of chronic diseases.

Based on observations in the past and present, we believe that it is important to investigate a possible involvement of intestinal bacteria and their toxins in the development of ADs in conjunction with the host's low immune function as summarized in Figure 5. Patients with low immune function are more susceptible not only to pathogenic intestinal bacteria but also to nonpathogenic commensal bacteria and their toxins, as well as dietary components such as mimic antigens and fats. Importantly, these environmental factors do not affect the host under normal conditions but may affect the global host health and further deteriorate the immune system under conditions such as immunosenescence, GI disorders, or stress. Therefore, to fully appreciate the etiology of ADs, it is important to acknowledge possible low immune function and relating minor disorders or defects, which are observed before the onset of ADs, such as RA.

## Figures and Tables

**Table 1:** Internal and exogenous factors possibly involved in the pathogenesis of RA and other ADs: two unrelated types of risk factors apparently contribute to the development of ADs: the first is environmental exogenous factors, and the second is the host's physical condition. In addition, ADs such as RA might be classified into two types, autoantibody-mediated and non-antibody-mediated. Regardless of whether the disease is mediated by autoantibodies or not, bacterial toxins absorbed from the intestine directly trigger and exacerbate an inflammatory reaction.

Types of risk factors	Antibody-mediated arthritis	Non-antibody-mediated arthritis
Exogenous factors	Possible risk agents	
Mimic antigens	Dietary collagen [29, 30], Cartilage PG [94]	/
Pathogenic and nonpathogenic bacteria	PAMPs [13, 14, 95]	PAMPs [13, 14, 95] Arthritogenic PG-PS [96]
Bacterial toxins	LPS [28, 97], MAM [28, 98], and SEB [99]	LPS [30], SEB [3]
Foods and drugs	High fat diet [90], Proteinase inhibitor, and NSAID [30]	High fat diet [90], Proteinase inhibitor, and NSAID [30]

Host's physical condition	Possible risk events	
Immune function	Immunosenescence [47, 49] GI disorders [44]	Immunosenescence [47, 49] GI disorders [44]
Mucosal barrier function	Stress [41, 42, 100] GI disorders [44]	Stress [41, 42, 100] GI disorders [44]
Intestinal bacterial balance	Bacterial imbalance [16, 18, 75] High toxin level in GI tract	Bacterial imbalance [16, 18, 75] High toxin level in GI tract
